# Soil–Plant–Water Systems and Interactions

**DOI:** 10.3390/plants13030358

**Published:** 2024-01-25

**Authors:** Ágota Horel

**Affiliations:** Department of Soil Physics and Water Management, Institute for Soil Sciences, HUN-REN Centre for Agricultural Research, Ruszti út 2–4, H-1022 Budapest, Hungary; horel.agota@atk.hun-ren.hu

## 1. Introduction

To comprehend the soil–plant–water system and how its constituents interact with each other, it is essential to better understand its effect on ecosystems. This knowledge helps in the preparation of mitigating actions, such as managing water and nutrients efficiently, optimizing crop productivity, enhancing biodiversity, or evaluating environmental impacts.

Soil ecosystems are complex and dynamic systems influenced by many factors, including soil–plant–water interactions and the exchange of carbon, nutrients, and water between soils, plants, and the atmosphere. Water is vital for plants. The amount of water present in the soil affects water and nutrient uptake by plants and the activity of soil microorganisms, while plants affect soil water dynamics via their root structure and density, water uptake requirements, or transpiration. The water balance is also influenced by both soil structure and plant presence due to factors such as different rates of evaporation or interception values. All these effects can be enhanced or decreased by changes in environmental conditions (e.g., meteorological or soil management changes) and therefore necessitate a comprehensive evaluation and understanding of mitigating measures.

Climate change is inevitable, resulting in changes in precipitation amounts and intensities, along with changes projected with respect to air temperature [1]. It affects the soil–water–plant system in many ways, and among them, one of the most important is drought conditions. Arid conditions, water deficiency, or irrigation causing plant stress are very current issues. Studies helping the better understanding of plant mechanisms and different stress conditions are necessary and crucial; therefore, climate-change-related research topics are going to be the main focus of many submitted papers in this Special Issue (SI).This SI was an invitation to researchers in the field who investigate part of or the entirety of the soil–plant–water system, and this SI received many papers on topics such as climate-change-related issues (e.g., arid conditions, water deficiency, irrigation), soil chemical parameters affecting soil water and plant health, inter-row soil management, over-harvesting, modeling crop water use, plant water uptake, and in general, sustainable agriculture.

## 2. Climate Change and Soil Water Deficits

One of the most important changes that our soil–plant–water system currently faces is drought conditions. Studying plant response to drought is necessary as it affects crop production, impairs plant growth and development, might reduce water-use efficiency, and in general helps us better estimate future climate change impacts on plant communities and their surrounding ecosystems. This topic is addressed in many studies or part of the studies presented in this SI.

Gao et al. [2] investigated medicinal plant growth, physiology, and antioxidant activity under drought stress. The paper focuses on the *Rabdosia rubescens* plant, which is a medicinal plant used for a variety of health concerns due to its high terpenoid, flavonoid, polysaccharide, and organic acid contents [2,3]. Due to drought stress, the plant showed a reduction in shoot growth, dry mass, and oridion, chlorophyll, and protein contents. The authors also investigated the effects of LED lighting and mowing effects on *R. rubescens*, but they observed no significant enhancements in the plant’s ability to cope with drought in their studied environment.

Climate change and the ability of plants to adapt to it was the focus of the paper published by Bochicchio et al. [4]. The plant of interest was the Saragolle lucana wheat (*Triticum durum* Desf), where the authors studied Saragolle root architecture, rhizosheath formation, and biomass allocation changes due to water shortage. The authors observed significantly smaller leaf areas; shorter heights with respect to the tip of the leaf; lower shoot dry mass, root mass, total root length, and root volume; and significantly higher rhizosheath mass, root tissue density, and seedling shoot dry matter concentrations for water-deficient plants. The authors conclude that the studied ancient wheat type has an interesting trait for plant performance in conditions of low soil water availability and for relations with the rhizosphere.

A different variety of wheat (*Triticum aestivum* L.) was investigated by Abakumov et al. [5], and they studied its ability to develop under different mineralization rates in irrigation water as a conservation agriculture technique. The three-year-long study aimed at finding a mineralization water rate condition in which wheat yield can be enhanced or even maximized, keeping in mind the danger of salinization or the alkalinization of irrigated soil. As clean surface water scarcity might be an issue in the near future and many locations, the authors presented this alternative technique of irrigating wheat with mineral water over the use of river water.

Saieed et al. [6] also focused their research on the changing climate and the ability of plants to adapt to it. The authors investigated the phenotypic plasticity of wheat in a double haploid population, evaluating the variation in yield-contributing traits; estimated traits’ plasticity and interrelation; and determined the basis of trait plasticity that is favored by grain yield or grain protein content. The experimental setup included different nitrogen fertilizer levels. The authors found that plasticity performances were environmentally dependent. Furthermore, their findings suggest that selecting for high-yield plasticity may be more effective with respect to improving crop yields than selecting for stress tolerance alone, as yield plasticity was lower under unfavorable conditions. High-yield plasticity is positively associated with the plasticity of seed length and number but negatively associated with the plasticity of grain protein content.

Arid conditions were the focus of a paper written by Bhanwaria et al. [7]. The authors aimed to study the stoichiometry of manure and moisture regimes on soil properties, microbial biomass C:N:P turnover, and the grain yield of mustard crops (*Brassica juncea* L.) under stress in arid conditions. The study showed that organic matter addition significantly increased moisture retention and the available water amount in soil, along with microbial biomass, electrical conductivity, capacity of cation exchange capacity, organic carbon, and saturated hydraulic conductivity, while reducing soil bulk density and pH. The paper emphasizes that based on soil amendments and irrigation water quality, the mustard grain and stover yield can be enhanced. The authors also highlight that the increasing levels of salinity in water reduces soil biological activity, such as turnover rate, build-up, or fluxes. This study helps understand the characteristics of the stoichiometric homeostasis of the soil microbial biomass of carbon, nitrogen, and phosphorus in an arid climate.

Modeling biomass production and crop water usage is a very important aspect of helping stakeholders prepare for changes in environmental conditions, such as prolonged drought periods. This topic was the focus of a study by De Barros et al. [8], where the authors investigated the spatial application of the AquaCrop model. The authors validated the model with field measurements and demonstrated a good correlation between the modeled and NDVI-based biomass of winter wheat; however, canopy cover estimations were more susceptible to other factors, such as the effect of different growth stages of winter wheat.

## 3. Forest, Shrubs, and Woody Plant Ecosystems

Forest, shrub, and woody plant ecosystems are natural systems mainly comprising trees (forest) and other plants, animals, and microorganisms. Because they produce oxygen and store carbon dioxide, they help regulate climate and help mitigate the effects of climate change. Unfavorable conditions, such as nutrient-deficient or highly degraded soils can induce stress response in plants, consequently influencing their health, development, and productivity.

Xiong et al. [9] investigated eight different forest communities for water holding capacity, species diversity, soil conservation, and carbon storage characteristics and their trade-off/synergies. The authors aimed to find methods for enhancing ecological functions and provide a scientific basis for sustainable regional development based on different indices for soil desiccation evaluation. The paper highlights the importance of significant differences among forest species diversity, soil, and plant carbon stock among forest communities and that there is a synergistic relationship between water-holding capacity and species diversity, carbon storage, and soil conservation. Therefore, based on ecological and biological community constituents, forest ecosystems need distinct evaluations when studying system function optimization.

Zhou et al. [10] investigated how afforestation, especially the age of different forest plants, affects soil moisture contents while focusing on plantations with desert shrubs or small trees (*Haloxylon ammodendron*). The authors have developed different indices for soil desiccation evaluation. Soil moisture to the depth of 400 cm was investigated under five distinct forest age groups. The manuscript highlights the importance of plant age and its water consumption, and it indicates the careful planning of afforestation with specific plants in terms of plant water usage. The authors also emphasize the need to implement water retention measures to mitigate soil water storage decline due to afforestation and consequently alleviate soil ecosystem degradation.

Horel and Zsigmond [11] investigated sloping vineyard sites under different inter-row soil management. The paper aims to study how different inter-row soil management methods (tillage, perennial grass, or cover crops) affect soil water content in and between grapevine rows and how vegetation indices such as NDVI, PRI, LAI, and fAPAR change in relation to inter-row management and slope position. The authors found that both inter-row management and slope position can significantly influence the physical and chemical parameters of soil that affect plant growth, and consequently, they can accelerate plant stress under sub-optimal environmental conditions, such as prolonged drought. The study found that the effects of climate change on hydrological soil processes can significantly affect soil–plant–water relations; therefore, longer-term monitoring is highly advised in affected areas.

Phosphorous soil is one of the major nutrients necessary for plants to grow. The study by Peleja et al. [12] showed how *Carapa guianensis* Aubl. respond to phosphorus addition based on biomass production, how the treatments affect the plants’ carbohydrate production, and how their dynamics change over different seasons. During the experimental period, the authors found no increase in dry matter content in the leaves and stems of the plant seedlings as a response to fertilizer treatments. Seasonality, however, showed differences in sugar amounts, reflecting the importance of plant responses to changing environmental conditions, such as during dry or wet periods.

*Capparis spinosa* L.’s growth at different sites was studied by Isagaliev et al. [13]. The authors characterized the cenopopulation dynamics and plant development patterns of *C. spinosa* L. Plant and soil samples were analyzed for elemental compositions. The study highlights how different growing conditions, such as soil texture or chemistry, can influence plant stem length, number of plants per area, or plant micronutrients.

## 4. Alternative Crop Production

Alternative crop production methods, such as hydroponics, aquaponics, or soilless vegetable production, are increasingly attracting attention due to their potential in enhancing sustainable agriculture.

Ding et al. [14] investigated the zero discharge cultivation of cucumbers (*Cucumis sativus* L.) under greenhouse conditions. The authors investigated various plant parameters, such as fruit quality and yield, leaf gas exchange parameters, chlorophyll content, general plant parameters (e.g., height and leaf area), and leaf mineral elements (e.g., N, Fe, Mn, B, and Cu). The study concluded that the zero discharge of nutrient solution treatment did not change cucumber growth parameters, photosynthesis, relative chlorophyll content, cucumber yield, and fruit quality; therefore, this can be an environmentally friendly technique as it can save water and fertilizer and reduce environmental pollution.

The topic of indoor vegetable production is the topic of the review paper by Ampim et al. [15], as accessibility with respect to growing vegetables can be limited in many places. The authors have reviewed many alternative vegetable production approaches, such as hydroponics, aeroponics, aquaponics, and soilless mixes. The review paper further describes amenities for alternative vegetation production, different plant growth factors, and challenges associated with indoor vegetable production, such as energy costs.

## 5. Conclusions and Future Perspectives

This SI included very diverse types of papers from many scientific disciplines. A significant amount of research work included environmental applications of nutrients (nitrogen or phosphorous) and studying specific plants (*Brassica juncea* L., *Haloxylon ammodendron* L., *Capparis spinosa* L., *Carapa guianensis* Aubl., *Cucumis sativus* L., *Triticum aestivum* L., *Triticum durum* Desf., *Vitis vinifera* L., and *Rabdosia rubescens* L.) or plant communities (e.g., forest ecosystems). We learned from this SI how important it is to study the soil–plant–water system as an interconnected system, as all parts have their vital functions. However, the authors acknowledge and address several unresolved issues and limitations in their studies, highlighting the need for further research. The necessity for longer field trials and additional study areas with different plant and/or soil types was also underlined in many studies.

Overall, this SI emphasizes the knowledge gaps and research priorities with respect to the interactions between biotic factors, such as plant mechanisms and plant health, and abiotic factors, such as soil physical and chemical properties, influencing soil health under varying environmental and management conditions.
